# miR-101-3p sensitizes non-small cell lung cancer cells to irradiation

**DOI:** 10.1515/med-2020-0044

**Published:** 2020-06-07

**Authors:** Zhonghui Li, Zhenjie Qu, Ying Wang, Meilin Qin, Hua Zhang

**Affiliations:** Department of Oncology, the Third Affiliated Hospital of Inner Mongolia Medical University, Inner Mongolia, China; Department of Gerontology, the Third Affiliated Hospital of Inner Mongolia Medical University, Inner Mongolia, China

**Keywords:** miR-101-3p, mTOR, NSCLC, irradiation

## Abstract

Recent studies have revealed that microRNAs regulate radiosensitivity of non-small cell lung cancer (NSCLC). The aim of this study was to investigate whether miR-101-3p is correlated with radiosensitivity of NSCLC. According to our results, miR-101-3p was downregulated in NSCLC tissues and cell lines. Moreover, miR-101-3p was decreased in A549 cells’ response to irradiation in a dose-dependent manner. Upregulation of miR-101-3p decreased survival fraction and colony formation rate and increased irradiation-induced apoptosis in irradiation-resistant cells, while miR-101-3p depletion had the opposite effects in irradiation-sensitive cells. Furthermore, mechanistic target of rapamycin (mTOR) is a target gene of miR-101-3p. The expressions of mTOR, p-mTOR, and p-S6 were curbed by overexpression of miR-101-3p in A549R cells, which was enhanced by repression of miR-101-3p in A549 cells. Intriguingly, elevation in mTOR abated miR-101-3p upregulation-induced increase in irradiation sensitivity in irradiation-resistant cell line. In contrast, rapamycin undermined miR-101-3p inhibitor-mediated reduction of irradiation sensitivity in irradiation-sensitive cell line. Besides, miR-101-3p overexpression enhanced the efficacy of radiation in an NSCLC xenograft mouse model. In conclusion, miR-101-3p sensitized A549 cells to irradiation via inhibition of mTOR-signaling pathway.

## 1 Introduction

Lung cancer is the leading cause of cancer-associated deaths worldwide, and non-small cell lung cancer (NSCLC) accounts for the majority of lung cancer cases [1]. Nowadays, radiotherapy is an important treatment option for NSCLC patients [2,3]. However, radioresistance is a biological behavior of cancer cells that limits the efficacy of radiotherapy [4]. For NSCLC patients with radiotherapy, further improvements in treatment outcome are likely to come from an understanding of radiotherapy at the molecular level.

MicroRNAs (miRNAs), small noncoding RNAs, are aberrantly expressed in a variety of cancer cells and play irreplaceable roles in regulating gene expressions, thereby modulating downstream signaling pathways and affecting cancer development and progression [5]. It is well-documented that miRNAs may be novel molecular biomarkers of biochemical recurrence for NSCLC patients and determine prognosis and response to therapy [6,7]. Some studies have revealed that radiation induces changes in miRNA expressions in NSCLC [8,9]. Given these points, we sought to find the correlation between radiosensitivity and dysregulation of miRNAs as well as their underlying molecular mechanisms. Recently, the role of miRNA-208a in NSCLC radioresistance has been identified, indicating that miRNAs may serve as potential radiotherapeutic targets [9]. miR-101-3p is involved in the progression of multiple cancers, including NSCLC [10,11,12]. It has been reported that miR-101-3p reverses temozolomide resistance by inhibiting glycogen synthase kinase 3β in glioblastoma [13]. In another study, miR-101-3p inhibits autophagy and enhances cisplatin-induced apoptosis of hepatoma cells [10]. Researchers also indicated that miR-101-3p blocks the phosphatidylinositol 3-kinase (PI3K)/AKT-signaling pathway by targeting metastasis associated lung adenocarcinoma transcript 1, leading to the inhibition of growth and metastasis of NSCLC [14]. Those data taken together suggest that miR-101-3p may act as a tumor suppressor gene in NSCLC. Additionally, it has also been reported that miR-101-3p increases the sensitivity of large cell lung cancer cell line 95C and neuroblastoma cell line U87 to radiation by targeting DNA-dependent protein kinase catalytic subunit and ataxia telangiectasia mutated (an important checkpoint regulator for promoting homologous recombination repair) [15]. Although the functional roles of miR-101-3p have been well described in lung cancer, the functional mechanisms of radiosensitivity in NSCLC are not yet fully elucidated.

Mechanistic target of rapamycin (mTOR) signaling is correlated with cell apoptosis in a variety of cancers [16,17]. Previous study has demonstrated that miR-145-3p suppresses cell growth, motility, and chemotaxis in NSCLC via suppressing the mTOR pathway [18]. More importantly, miR-101-3p could regulate proliferation and apoptosis of human osteosarcoma cells by targeting mTOR [19]. However, whether miR-101-3p is associated with radiosensitivity by targeting mTOR in NSCLC needs to be further explored. The purpose of this study was to examine the roles of miR-101-3p and mTOR in radiosensitivity of NSCLC as well as their downstream signaling pathways. In this study, we demonstrated that miR-101-3p sensitized A549 cells to irradiation via inhibition of mTOR-signaling pathway by performing quantitative real-time polymerase chain reaction (qRT-PCR), colony formation, flow cytometry, Western blot, luciferase reporter, and tumor xenograft *in vivo* assays.

## 2 Materials and methods

### 2.1 Clinical samples and cell culture

NSCLC tissues and adjacent normal tissues (NTs) were obtained from 35 NSCLC patients at the Third Affiliated Hospital of Inner Mongolia Medical University. Written informed consent was signed by patients or their relatives prior to this study. Study approval was obtained from the Research Ethics Committee of the Third Affiliated Hospital of Inner Mongolia Medical University. The correlation between miR-101-3p expression and clinicopathological features of NSCLC patients (35 cases) is displayed in Supplementary Table 1. Human NSCLC cell lines A549, H520, and H460 and human bronchial epithelial cell line 16-HBE were purchased from American Type Culture Collection (Manassas, VA, USA). Cells were cultured in Dulbecco’s modified Eagle’s medium (Thermo Fisher Scientific, Waltham, MA, USA) supplemented with 10% of fetal bovine serum (Thermo Fisher Scientific) and 1% of penicillin/streptomycin stock solution (Sigma, St. Louis, MO, USA). All cells were incubated at 37°C with 5% CO_2_.

### 2.2 Establishment of irradiation-resistant cell lines

To explore the expression of miR-101-3p in NSCLC cell lines’ response to irradiation, A549 cells were first grown to approximately 90% confluence and then were irradiated with doses ranging from 0 to 8 Gy X-irradiation. Following X-irradiation, culture medium was replaced with fresh medium and the cells were returned to a 37°C incubator for further growth. Irradiation dosage of 4 Gy was chosen as the standard for the following experiments. To generate irradiation-resistant cells, A549 cells (90% confluence) were irradiated with 2.0 Gy/fraction using 6 MV X-rays generated by an accelerator provided by the Third Affiliated Hospital of Inner Mongolia Medical University, and the final doses were 64 Gys. The selected radioresistant cell line was named A549R.

### 2.3 Cell transfection and treatment

A549R or A549 cells were transfected with miR-101-3p mimic (miR-101-3p), negative control mimic (miR-NC), miR-101-3p inhibitor (anti-miR-101-3p), negative control inhibitor (anti-miR-NC), mTOR overexpression plasmid (mTOR), or pcDNA 3.0 vector (vector) using Lipofectamine 3000 (Thermo Fisher Scientific). To inhibit the mTOR-signaling pathway, A549R or A549 cells were treated with rapamycin (Sigma). Rapamycin was dissolved in dimethyl sulfoxide (Sigma) at a concentration of 1 mM and stored at −20°C, which was diluted to the appropriate concentration in the serum containing the culture medium just before addition to cell cultures at a final concentration of 0.01% of the vehicle.

### 2.4 qRT-PCR

Total RNAs were extracted from cells using Trizol reagent (Sigma) and reversely transcribed into complementary DNA using TaqMan® MicroRNA Reverse Transcription kit (Biosystems, Foster City, CA, USA). qPCR was performed using SYBR® Green (Promega, Madison, WI, USA). Primers were listed as follows: miR-101-3p forward, 5′-GCCGCCACCATGGTGAGCAAGG-3′ and reverse, 5′-AATTGAAAAAAGTGATTTAATTT-3′; and U6 forward, 5′-GCTTCGGCAGCACATATACTAAAAT-3′ and reverse, 5′-CGCTTCACGAATTTGCGTGTCAT-3′. The relative level of miRNA (normalized to U6 small nuclear RNA) was analyzed by the 2^−ΔΔCt^ method [20].

### 2.5 Colony formation assay

The survival fraction was determined using colony formation assays. Cells were irradiated with 0, 2, 4, 6, and 8 Gy X-irradiation and then incubated for 14 days. The colonies were fixed with 4% paraformaldehyde (Sigma) for 15 min and stained with 1% crystal violet (Beyotime, Shanghai, China) for 10 min. The number of colonies was counted in five randomly chosen fields and microscopic colonies containing >50 cells were counted as having arisen from single surviving cells. The survival fraction was calculated as (number of colonies/number of cells plated)_irradiated_/(number of colonies/number of cells plated)_non-irradiated_. Each group was conducted with three replicates.

### 2.6 Cell apoptosis assay

A549R or A549 cells were trypsinized, collected, and washed with phosphate buffer solution (PBS). Cell apoptosis was analyzed using FITC annexin V apoptosis detection kit (BD Biosciences, Franklin Lakes, NJ, USA). Cells were labeled with 5 µL of annexin V-FITC and 5 µL of propidium iodide and kept in the dark for 15 min at room temperature. Cell apoptotic rate was detected by an FACSCalibur flow cytometer with Cell Quest software (BD Biosciences).

### 2.7 Western blot

Cells were treated with RIPA buffer (Thermo Fisher Scientific) and quantified with the Bio-Rad protein assay kit (Bio-Rad Labs, Richmond, CA, USA). Equal amounts of proteins per sample were separated by sodium dodecyl sulfate–polyacrylamide gel (Thermo Fisher Scientific) electrophoresis and transferred onto polyvinylidene fluoride membranes (Roche, Mannheim, Germany). Membranes were blocked with 5% skim milk in PBS containing Tween-20 (PBST) for 1 h at room temperature. The membranes were then incubated overnight at 4°C with primary antibodies (rabbit polyclonal anti-phospho-mTOR, rabbit monoclonal anti-phospho-S6; Abcam, Cambridge, MA, USA), followed by incubation of the horseradish peroxidase-conjugated secondary antibody (Abcam). The protein signals were visualized using enhanced chemiluminescence (Thermo Fisher Scientific). Densitometry values were normalized to levels of β-actin and analyzed by ImageJ software (US National Institutes of Health, Bethesda, MD, USA).

### 2.8 Luciferase reporter assay

The binding sites between miR-101-3p and mTOR were predicted through Targetscan online database. The luciferase reporter plasmids containing the wild-type (Wt) or mutated (Mut) miR-101-3p binding sites in the 3′-UTR of mTOR were constructed. mTOR-Wt or mTOR-Mut and miR-101-3p mimic, inhibitor, or their negative controls were transfected into A549R or A549 cells using Lipofectamine 3000. Cells were harvested at 48 h after transfection. The luciferase activity was measured using the Dual Luciferase Reporter Assay System (Promega) according to instructions.

### 2.9 Tumor xenografts *in vivo*

The experiments were approved by the animal care and experiment committee of the Third Affiliated Hospital of Inner Mongolia Medical University. The 18 female BALB/c nude mice (20 to 22 g, 4 to 6 weeks) were purchased from the Shanghai Laboratory Animal Center (Shanghai, China). Seven days after A549 cell injection in mice (*n* = 6), miR-101-3p mimic or negative control mimic was intratumorally injected into the implanted tumor every 3 days for seven times. Mice were irradiated with 4 Gy once per day for the following 5 days. Tumor volumes were measured every 3 days after injection. Mice were euthanized at 28 days after injection of cells and tumor weight was measured. qRT-PCR and Western blot were performed to detect the expressions of miR-101-3p and mTOR in tumor tissues of mice, respectively.

### 2.10 Statistical analyses

Data are presented as mean ± standard deviation. All statistical analyses were performed using SPSS 18.0 software (SPSS, Inc., Chicago, IL, USA). The differences were evaluated by using Student’s *t* test or one-way analysis of variance. Values of *P* < 0.05 were considered statistically significant.

## 3 Results

### 3.1 miR-101-3p decreased in NSCLC tissues and cells treated with irradiation

**Figure 1:**
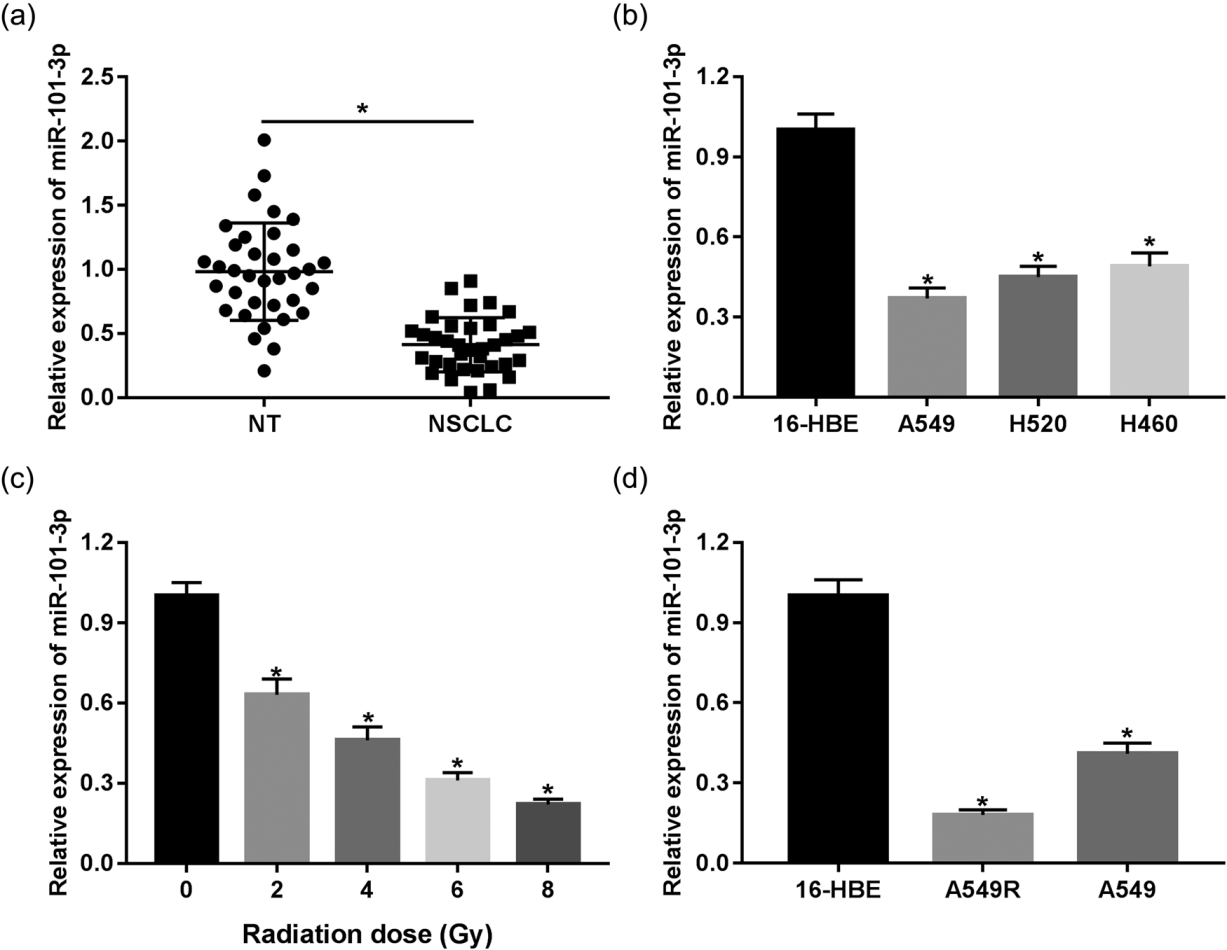
miR-101-3p is decreased in NSCLC tissues and cells treated with irradiation. (a) qRT-PCR was performed to measure the expression of miR-101-3p in tissues of 35 patients with NSCLC. (b) The expression of miR-101-3p in human NSCLC cell lines A549, H520, or H460 and human bronchial epithelial cell line 16-HBE. (c) The level of miR-101-3p in A549 cells irradiated with doses ranging from 0 to 8 Gy X-irradiation. (d) The level of miR-101-3p in 16-HBE, A549, and A549R. *P < 0.05.

First, we obtained NSCLC tumor tissues and NTs from 35 patients without any treatment except surgery. miR-101-3p was found to be significantly downregulated in NSCLC tissues compared with that in NTs (Figure 1a). In addition, we also found that low miR-101-3p expression was associated with tumor node metastasis (TNM) stage and lymph node metastasis (Supplementary Table 1). Subsequently, we analyzed the expression levels of miR-101-3p in 16-HBE human bronchial epithelial cell line and NSCLC cell lines. The result of qRT-PCR showed that miR-101-3p expression was notably lower in A549, H520, and H460 cells than that of 16-HBE cells (Figure 1b), which was consistent with that in tissues. To further explore the role of miR-101-3p in irradiation sensitivity of human NSCLC, the expression levels of miR-101-3p in A549 cells were measured following irradiation treatment of 0 to 8 Gy. Our results showed that miR-101-3p was reduced in A549 cells’ response to irradiation in a dose-dependent manner (Figure 1c). We found that A549 cells with 4 Gy irradiation dosage had 35% of survival fraction, while A549R with 4 Gy irradiation dosage had 70% of survival fraction. In addition, the irradiation treatment of 2 Gy had less effect on the two cells. Meanwhile, cell survival was poor in A549 and A549R cells after 6 Gy irradiation treatment. Therefore, 4 Gy dose was selected as the treatment dose. Additionally, an irradiation-resistant cell line was established from A549 cells, namely, A549R cells. Interestingly, the expression of miR-101-3p in irradiation-resistant A549R cells was observed to be lower than that of irradiation-sensitive A549 cells (Figure 1d), indicating that miR-101-3p may be implicated in the regulation of irradiation sensitivity.

### 3.2 miR-101-3p sensitizes A549 cells to irradiation

**Figure 2:**
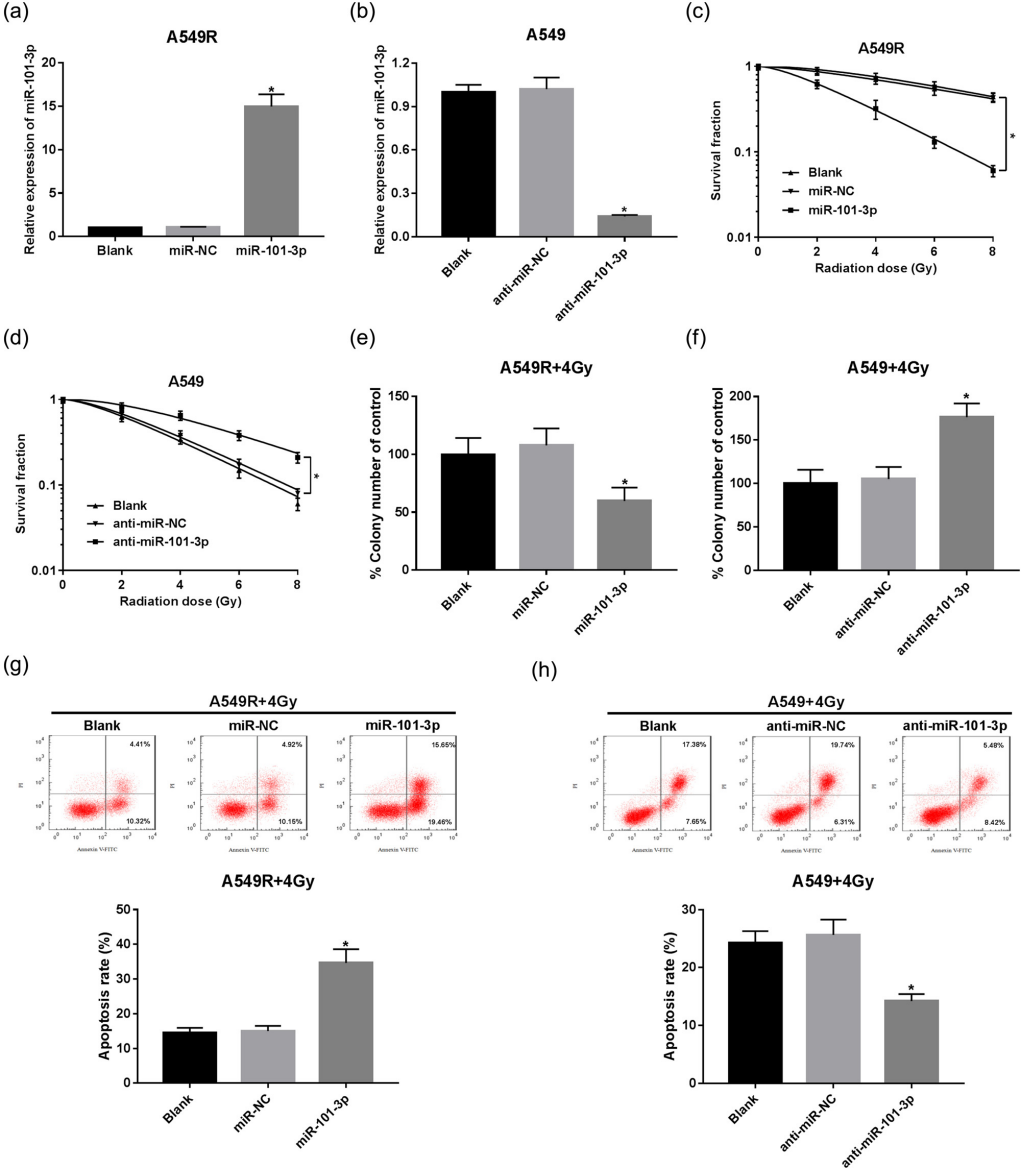
miR-101-3p sensitizes A549 cells to irradiation. (a) A549R cells were transfected with miR-NC or miR-101-3p mimic and qRT-PCR was used to examine the level of miR-101-3p. (b) The level of miR-101-3p in A549 cells transfected with anti-miR-NC or miR-101-3p inhibitor. (c and d) The survival fraction was determined using colony formation assay in A549R and A549 cells. (e and f) Colony formation rate was calculated in irradiation-treated A549R or A549 cells transfected with miR-NC, miR-101-3p mimic, anti-miR-NC, or miR-101-3p inhibitor. (g and h) Cell apoptosis rate was evaluated in A549R and A549 cells using flow cytometry. *P < 0.05.

Furthermore, we sought to address the effect of miR-101-3p on irradiation sensitivity of NSCLC cells. A549R cells were transfected with miR-101-3p mimic or miR-NC. By contrast, A549 cells were transfected with miR-101-3p inhibitor or anti-miR-NC. The transfection of miR-101-3p mimic was efficient to induce higher expression of miR-101-3p in irradiation-resistant cells, while miR-101-3p inhibitor introduction reduced the level of miR-101-3p in irradiation-sensitive cells (Figure 2a and b). Then, A549R, A549, or transfected cells were exposed to various doses of irradiation (0, 2, 4, 6, or 8 Gy). We found that miR-101-3p enhanced the effect of irradiation in A549R cells, whereas the block of miR-101-3p decreased its effect in A549 cells (Figure 2c–f). Radiosensitivity is often related to cell apoptosis. Therefore, cell apoptosis rate was evaluated using flow cytometry. The results showed that miR-101-3p combined with irradiation demonstrated a higher percentage of cells in apoptosis than that of the miR-NC group in A549R cells (Figure 2g), while miR-101-3p depletion combined with irradiation significantly decreased the ratio of apoptotic A549 cells (Figure 2h). These data suggested that miR-101-3p could increase the irradiation sensitivity of NSCLC cells.

### 3.3 mTOR is a direct target of miR-101-3p

**Figure 3:**
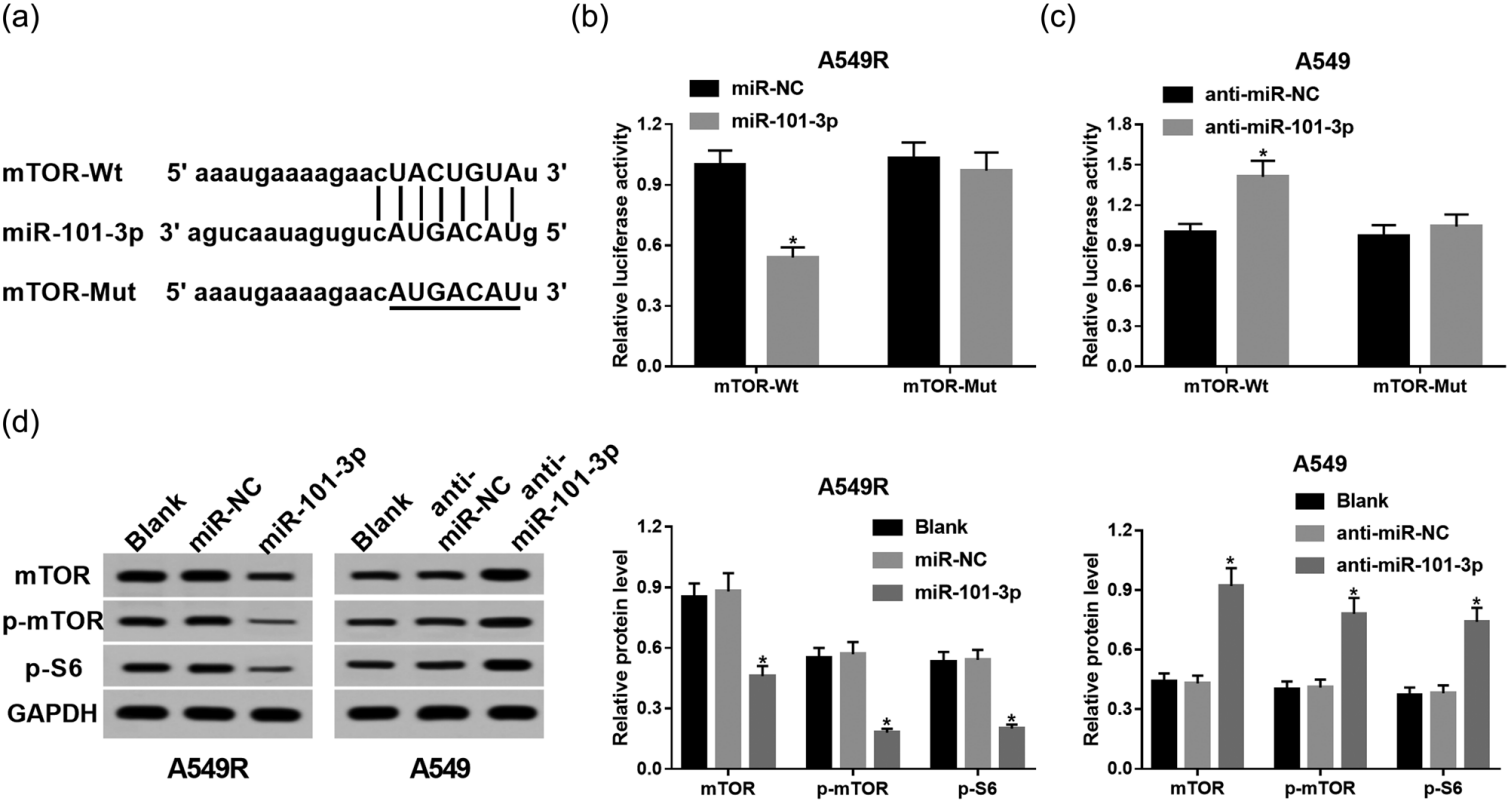
mTOR is a direct target of miR-101-3p. (a) The binding sites between miR-101-3p and mTOR were predicted by Targetscan online database and the luciferase reporter plasmids containing the wild-type or mutated mTOR binding sites of miR-101-3p were established. (b) The luciferase activity was examined in A549R cells cotransfected with mTOR-Wt or mTOR-Mut luciferase reporter and miR-101-3p mimic or miR-NC. (c) The luciferase activity was measured in A549 cells cotransfected with mTOR-Wt or mTOR-Mut luciferase reporter and miR-101-3p inhibitor or anti-miR-NC. (d) Western blot was conducted to detect the expression of mTOR, p- mTOR, and p-S6. *P < 0.05.

As miRNAs exert their roles through inhibiting target mRNA translation, identification of miR-101-3p target genes is a key step for understanding the mechanism of miR-101-3p – regulating NSCLC radiosensitivity. In this research, mTOR was predicted to contain binding sites with miR-101-3p using Targetscan online database (Figure 3a), indicating that mTOR may function as a target for miR-101-3p. To validate our speculation, the luciferase reporter plasmids for mTOR containing the Wt or Mut sites of miR-101-3p were generated. The introduction of miR-101-3p greatly decreased the luciferase activity in A549R cells transfected with mTOR-Wt, while no significant effect was observed in cells transfected with mTOR-Mut (Figure 3b). Moreover, miR-101-3p inhibitor increased the luciferase activity in the mTOR-Wt group compared with that in the mTOR-Mut group of A549 cells (Figure 3c). In addition, the protein levels of mTOR and components of its downstream pathway, including p-mTOR and p-S6, were subsequently examined. After transfection with miR-101-3p mimic, the levels of mTOR, p-mTOR, and p-S6 were decreased in A549R cells. In contrast, miR-101-3p inhibitor introduction promoted their abundances in A549 cells (Figure 3d).

### 3.4 mTOR participates in miR-101-3p-mediated irradiation sensitivity of A549 cells

**Figure 4:**
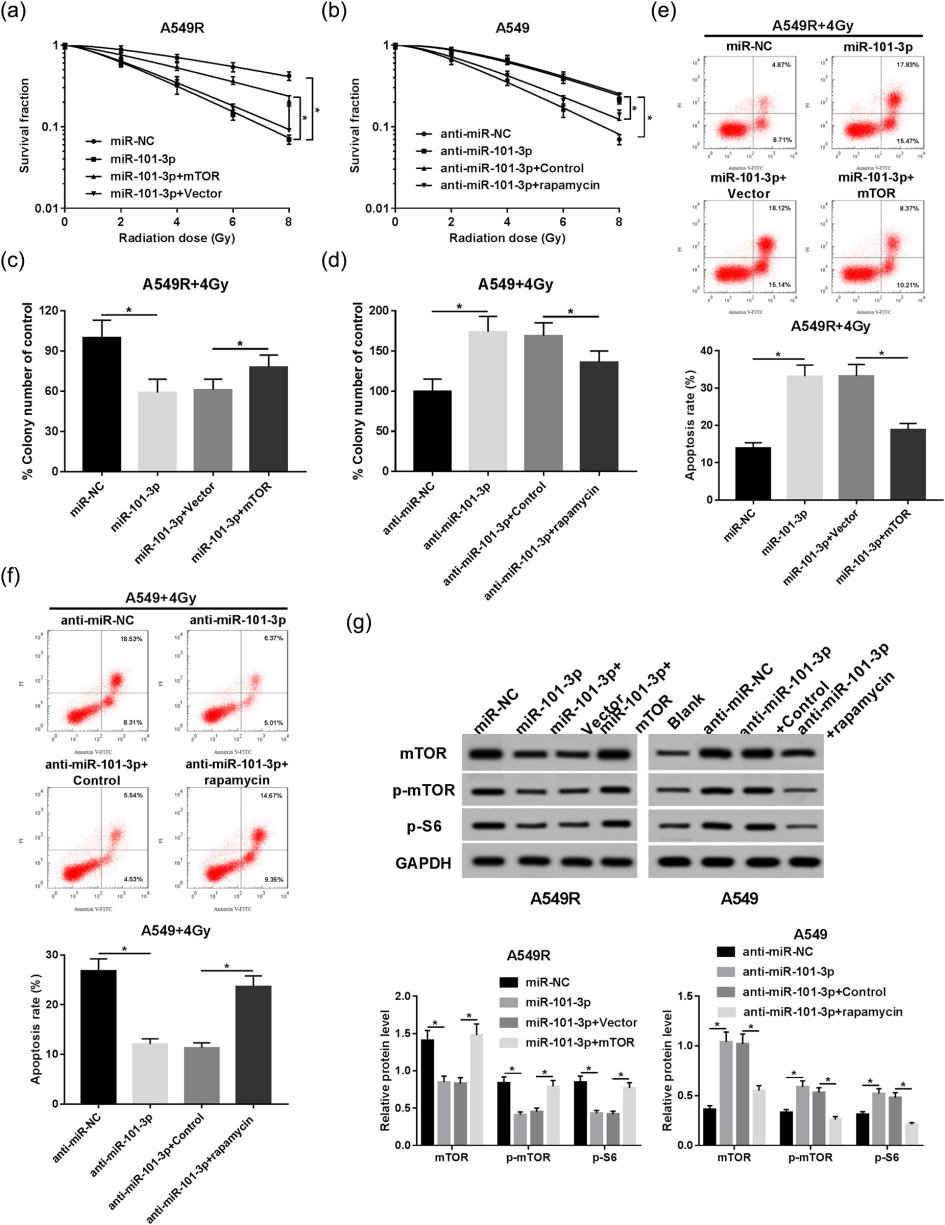
mTOR participates in miR-101-3p-mediated irradiation sensitivity of A549 cells. (a) A549R cells were introduced with miR-NC, miR-101-3p mimic, miR-101-3p mimic + mTOR overexpression plasmid, or miR-101-3p mimic + pcDNA vector; and the survival fraction was determined using colony formation assay. (b) A549 cells were transfected or treated with anti-miR-NC, miR-101-3p inhibitor, miR-101-3p inhibitor + control, or miR-101-3p inhibitor + Rapamycin; and the survival fraction was determined using colony formation assay. (c and d) The transfected or rapamycin-treated A549 and A549R cells were treated with 4 Gy irradiation, and the colony formation rate was calculated. (e and f) Cell apoptosis rate was analyzed in A549R and A549 cells using flow cytometry. (g) Western blot was used to evaluate the expression of mTOR, p-mTOR, and p-S6 in A549 and A549R cells. *P < 0.05.

Given that mTOR is a target of miR-101-3p, we sought to further investigate whether mTOR is responsible for miR-101-3p-mediated radiosensitivity of A549 cells. In this study, when mTOR overexpression plasmid was transfected into miR-101-3p upregulation A549R cells, the cells became more resistant to irradiation than control cells. By contrast, after mTOR was inhibited in miR-101-3p inhibitor transfected A549 cells by rapamycin, the cells became more sensitive to irradiation compared to the control cells (Figure 4a–d). Moreover, miR-101-3p promoted irradiation-induced apoptosis in A549R cells, which was abated by upregulation of mTOR (Figure 4e). In contrast, rapamycin attenuated the miR-101-3p depletion-mediated apoptosis inhibition in irradiation-treated A549 cells (Figure 4f). Western blot analysis of mTOR, p-mTOR, and p-S6 was performed to further test the role of mTOR signaling in this process. The results showed that miR-101-3p decreased the expression of mTOR signaling-related proteins in A549R cells, which were rescued by mTOR overexpression. Meanwhile, rapamycin undermined the miR-101-3p inhibitor-mediated promotion of mTOR signaling in A549 cells (Figure 4g).

### 3.5 miR-101-3p enhances irradiation sensitivity *in vivo*

**Figure 5:**
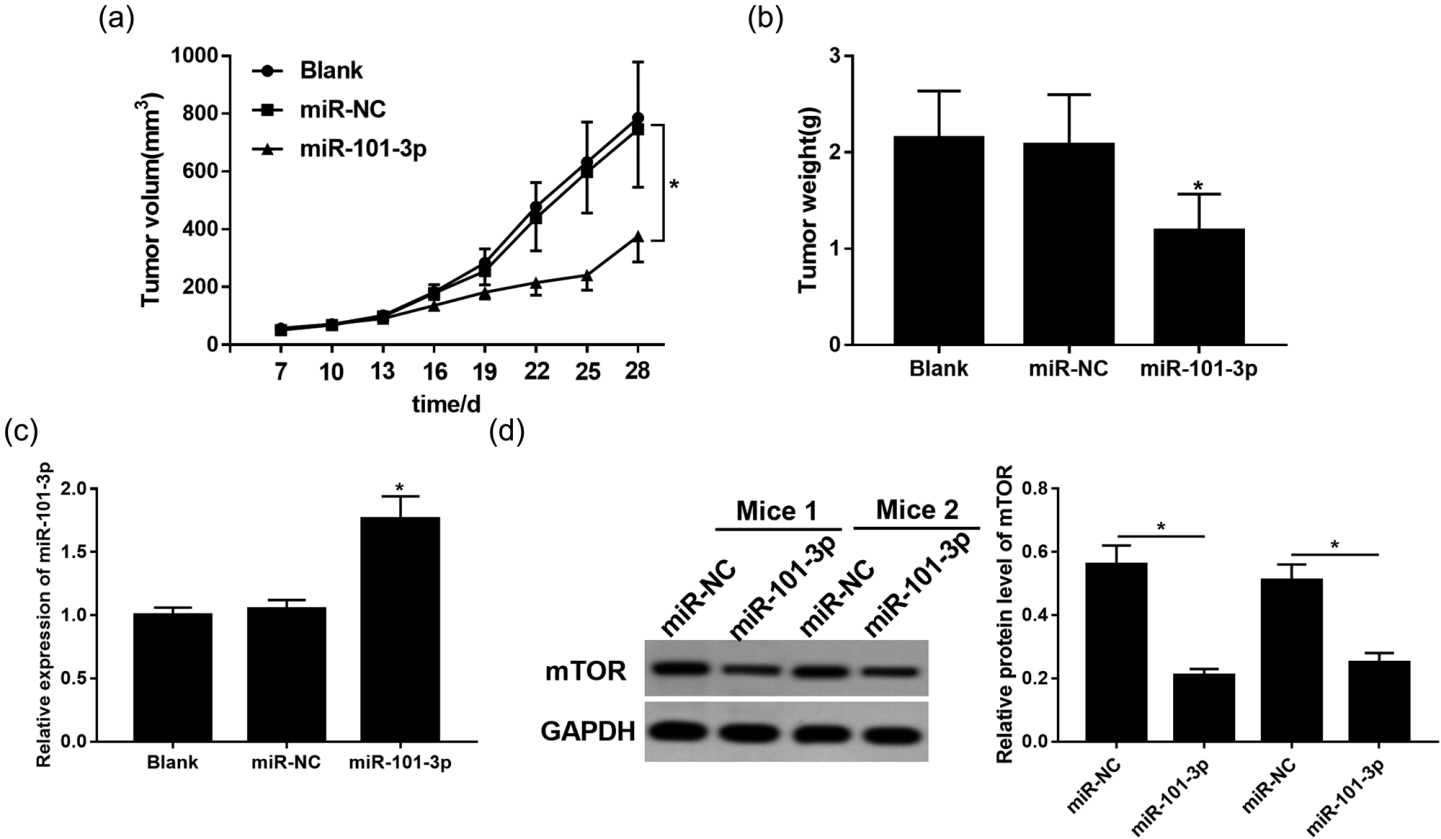
miR-101-3p enhances irradiation sensitivity *in vivo*. (a) Tumor volume was measured every 3 days after A549 cell injection. (b) Mice were euthanized at 28 days after cells injection and tumor weight was measured. (c) The level of miR-101-3p in mice injected with miR-101-3p mimic or miR-NC. (d) The expression of mTOR in mice injected with miR-101-3p mimic or miR-NC. *P < 0.05.

Based on the results that miR-101-3p was involved in irradiation sensitivity *in vitro*, we further validated the role of miR-101-3p *in vivo* using a mouse NSCLC model. The results showed that miR-101-3p combined with irradiation dramatically repressed tumor volume and weight compared with that in the miR-NC group (Figure 5a and b). Moreover, qRT-PCR and Western blot revealed that miR-101-3p was upregulated, whereas mTOR was inhibited in the miR-101-3p group (Figure 5c and d). These *in vivo* findings further proved that miR-101-3p enhanced the irradiation sensitivity of NSCLC by targeting mTOR.

## 4 Discussion

Radioresistance is the main obstacle for the current clinical management of NSCLC [21]. Therefore, preventing, predicting, and reducing NSCLC radioresistance are critical for further improving the survival rate of patients with NSCLC. Investigating the role of miRNAs in radiosensitivity is a promising avenue, given their ability to regulate multiple oncogenic processes including response to therapy. Recently, dysregulation of miRNAs is found to be associated with radiosensitivity of NSCLC [22,23]. miR-101-3p has been reported to be abnormally expressed in NSCLC and functioned as a tumor suppressor [14]. Moreover, miR-101-3p is evidently downregulated in NSCLC and is crucial for the increase in chemosensitivity to *cis*-diamminedichloroplatinum [24]. In the present study, miR-101-3p was decreased in NSCLC tissues. Lymph node metastasis is the most common metastasis pathway of NSCLC and one of the most important factors affecting prognosis and stage [25]. Comprehensive and accurate detection of the pathological state of lymph nodes can assist in accurate performance of postoperative TNM staging, guiding treatment, and prognosis, which are important for improving lung cancer survival rate and improving patients’ quality of life. Previous studies also suggest that miR-101-3p is related to metastasis of many cancers [26,27]. Our data also suggest that low miR-101-3p expression is associated with TNM stage and lymph node metastasis. However, the underlying mechanisms of how miR-101-3p expression regulates lymph node metastasis are not fully clear. Meanwhile, the A549, H520, H460, and 16-HBE cells have endogenous expression of miR-101-3p. Moreover, our result of qRT-PCR disclosed that miR-101-3p expression was notably lower in A549, H520, and H460 cells than that of 16-HBE cells, which is consistent with previous studies [14,24]. Moreover, miR-101-3p is reported to be involved in radioresistance of nasopharyngeal carcinoma [28]. However, it is still unclear whether the decrease in miR-101-3p in NSCLC regulates radiosensitivity. Therefore, we examined the changes in the level of miR-101-3p in irradiation-sensitive and irradiation-resistant NSCLC cells and found that miR-101-3p was decreased in irradiation-resistant cells, suggesting that miR-101-3p may be crucial in sensitizing cells to irradiation. Furthermore, we disclosed that miR-101-3p correlated with NSCLC cell survival and apoptosis. Overexpression of miR-101-3p increased radiosensitivity, whereas inhibition of miR-101-3p resulted in radioresistance.

Targetscan online database can predict many target genes (including mTOR) of miR-101-3p. Previous studies have revealed that rapamycin can increase the radiation sensitivity of lung cancer cells by inhibiting mTOR [29], and PI3K/mTOR inhibitor NVP-BEZ235 can enhance the radiosensitivity of human glioma stem cells [30]. mTOR is implicated in radiosensitivity of prostate cancer [31]. More importantly, mTOR has been reported to be the target of miR-101-3p in gastric cancer and vascular endothelial cells [32,33]. Those findings suggest that mTOR may be a potential oncogene and closely related to cell radiosensitivity. Hence, we hypothesized that miR-101-3p may increase the sensitivity of NSCLC to radiation exposure by targeting inhibition of mTOR expression and carried out our systematic research. In this study, miR-101-3p negatively regulated the expression of mTOR, suggesting that miR-101-3p may be responsible for radiosensitivity of NSCLC by inhibiting mTOR. Radiosensitivity is affected by a complex signaling cascade. The activation of mTOR-signaling pathway includes mTOR kinase itself and its downstream target, ribosomal protein S6 [34,35]. We further demonstrated that the expressions of mTOR pathway-related protein p-mTOR and p-S6 were reduced by overexpression of miR-101-3p, which were upregulated in the miR-101-3p depletion group of A549 cells. In addition, inhibition of mTOR pathway enhanced radiosensitivity in radioresistant prostate cancer cells via repressing colony formation, inducing more apoptosis, and reducing autophagy [36]. Rapamycin specifically inhibits the activity of mTOR [37,38]. For example, rapamycin increases the radiation-induced apoptosis and enhances the cytotoxic effect of radiation in NSCLC cell line H1299 [29]. Those data suggest that mTOR signaling may be an oncogene and lead to radioresistance. Consequently, it was studied whether mTOR signaling participates in the miR-101-3p-mediated radiosensitivity and apoptosis in NSCLC cell line A549.

Our findings showed that overexpression of mTOR displayed a counter-phenomenon with the effect of miR-101-3p overexpression on radiosensitivity of NSCLC. Meanwhile, it was also demonstrated that mTOR inhibition by rapamycin rescued the inhibition of radiosensitivity and apoptosis induced by miR-101-3p depletion. Those results confirmed that inactivation of mTOR signaling was a contributing factor to increased irradiation sensitivity in the induction of apoptosis, and miR-101-3p sensitized A549 cells to irradiation by blocking the mTOR-signaling pathway. Comprehensive research was also introduced to address the role of miR-101-3p in radiosensitivity *in vivo*, validating that miR-101-3p exacerbates the inhibition of tumor growth induced by irradiation in mice, which was consistent with the data *in vitro*. In addition, miR-101-3p also inhibited the expression of mTOR *in vivo*.

However, there are some limitations in this study. First, the mice NSCLC model where radioresistance was measured 28 days after treatments does not fully represent the true situation where patients usually develop radioresistance after months or years of first radiation. Our results *in vitro* have suggested miR-101-3p-mediated radiosensitization in NSCLC. The animal experiments in this study aimed to confirm the results of *in vitro* cell experiments and further explored whether miR-101-3p can exert radiosensitization *in vivo*, which may hardly be used as an animal model to simulate clinical issues completely. To the best of our knowledge, currently no good animal models are available to simulate this clinical process. However, previous studies have shown that multiple miRNAs can play roles in radiosensitization in lung cancer, such as miR-208a [9], miR-99a [39], and miR-148b [40], indicating that miR-101-3p is not unique. We did not evaluate the roles of other miRNAs in miR-101-3p-mediated irradiation sensitivity in NSCLC. Moreover, this report only investigated that miR-101-3p/mTOR signaling affects the radiosensitivity of NSCLC via modulating apoptosis, and the other molecular mechanisms underlying the miR-101-3p-mediated radiosensitivity of NSCLC will be determined in the future.

## 5 Conclusion

Taken together, our data highlighted the pivotal role of miR-101-3p in increasing the radiosensitivity of NSCLC *in vitro* and *in vivo*. We demonstrated that miR-101-3p could increase the irradiation-induced apoptosis, repress colony formation, and decrease survival fraction through inhibiting the mTOR-signaling pathway. A novel axis, miR-101-3p/mTOR signaling, was first observed to be responsible for radiosensitivity of NSCLC, which will enhance the efficacy of irradiation therapy and improve the survival of NSCLC patients.
